# The effect of a series of whole-body cryotherapy treatments on selected balance indicators in young, healthy people

**DOI:** 10.3389/fphys.2026.1871281

**Published:** 2026-07-23

**Authors:** Bartłomiej Ptaszek, Natalia Solecka, Szymon Podsiadło

**Affiliations:** 1 Institute of Applied Sciences, University of Physical Culture in Krakow, Krakow, Poland; 2 Faculty of Motor Rehabilitation, University of Physical Culture in Krakow, Krakow, Poland; 3 Institute of Clinical Rehabilitation, University of Physical Culture in Krakow, Krakow, Poland

**Keywords:** center of pressure, postural control, stabilometry, static balance, whole-body cryotherapy

## Abstract

**Background/objectives:**

Whole-body cryotherapy (WBC) is a physiotherapeutic method widely used in sports medicine, rehabilitation and biological regeneration. Its effects include modulation of the neuromuscular system, regulation of muscle tone and activation of adaptive mechanisms. Studies show that for people struggling with balance disorders, cryotherapy can reduce muscle spasticity and fatigue, often producing better results when combined with physical therapy. The aim of our study was to assess the effect of a series of whole-body cryotherapy treatments on selected parameters of static balance in healthy and young women, which also seems to be important.

**Methods:**

Twelve healthy young women participating in recreational physical activity participated in the study. Balance was assessed (before and after a series of 10 WBC sessions) using a stabilometric platform, which analyzed selected balance indicators. Measurements were performed while standing on one and both legs, with eyes open and closed.

**Results:**

Statistically significant changes were observed in selected stabilometric parameters. The most pronounced effects included a reduction in COP path length and mean velocity of COP displacement (COP sway path length (mm) BF EO; mean COP displacement velocity (mm/s) BF EO; average COP position in the X axis (mm) LL EO; average COP position in the Y axis (mm) LL EO), particularly during single-leg stance with eyes closed (COP oscillation path length (mm) LL EC; average COP displacement velocity (mm/s) LL EC; maximum COP oscillation (mm) LL EC; average COP position in the X axis (mm) RL EC). Changes were also noted in maximum sway and foot load distribution (loading of the left forefoot (%) BF EC; loading of the left hindfoot (%) BF EC).

**Conclusions:**

The use of a series of WBC treatments was associated with changes in selected stabilometric parameters, particularly under conditions of increased proprioceptive load. The results suggest a possible effect of WBC on postural control mechanisms in young and healthy people, but these require confirmation in randomized, controlled trials.

## 1 Introduction

Whole-Body Cryotherapy (WBC) is a physical therapy method that involves briefly exposing the patient’s entire body to extremely low temperatures in a chamber designed for such treatments. The temperature inside the cryochamber typically ranges from -110 °C to -160 °C, and the exposure lasts from 1 to 3 minutes. It creates a strong physical stimulus that triggers complex adaptive responses throughout the body (Bouzigon et al., 2016). One of the primary mechanisms of action of WBC is a rapid reduction in skin temperature, which results in vasoconstriction in peripheral tissues. This leads to a temporary reduction in peripheral blood flow and reduced metabolic processes. After the end of cold exposure, reactive vasodilation occurs. This phenomenon promotes improved microcirculation and influences the regulation of metabolic processes (Kim et al., 2014).

Exposure to low temperatures (local cryotherapy) slows nerve impulse conduction and reduces the excitability of sensory receptors. This mechanism is responsible for the analgesic effect of cryotherapy and influences the regulation of muscle tone and neuromuscular control (Algafly and George, 2007). These changes may have functional significance in the context of postural mechanisms, which largely depend on the efficient processing of proprioceptive stimuli. Research indicates that the effects of WBC are not limited to local effects but are systemic in nature. It has been demonstrated that a series of WBC treatments can influence the activity of the autonomic nervous system, leading to changes in the balance between sympathetic and parasympathetic activity. Of particular importance are the observations indicating the occurrence of the phenomenon of body adaptation after a series of cryotherapy treatments, which manifests itself in a weakened stress response to cold and stabilization of autonomic parameters. This observation also justifies examining the effects of a series of treatments rather than a single WBC session (Ziemann et al., 2012; Lombardi et al., 2017). The results of the study by Hausswirth et al. (2013) show that both partial and whole-body cold stimulation effectively stimulate the autonomic nervous system, with a predominance of parasympathetic activation, and also suggest that whole-body cold exposure induced greater autonomic nervous system stimulation compared to partial-body cold exposure (Hausswirth et al., 2013).

Static balance is defined as the body’s ability to maintain a stable body position with a stationary base of support or with minimal center of gravity displacement, without shifting the support area. Maintaining it requires the proper coordination of the nervous system, muscles, and sensory organs, and its proper functioning determines effective postural control in everyday activities (Horak, 2006). Maintaining a stable posture requires constant monitoring of the body’s center of mass relative to the support surface and generating appropriate postural corrections. This process occurs automatically and depends on the efficient integration of sensory information and an effective motor response (Winter et al., 1998).

Unfortunately, few studies have been published on the effects of cryotherapy on balance, and these studies concern local cryotherapy. Douglas et al. (2013) examined static and dynamic conditions and found a statistically significant increase only in the mediolateral component of the dynamic balance test after immersion in ice water. Williams et al. (2013) conducted two balance tests. They observed no difference after local cooling. Dewhurst et al. (2007) assessed two quiet standing positions with a large and narrow base of support, with eyes open and closed. Cooling both legs until muscle temperature dropped by 3 °C had no effect on postural assessment. Surenkok et al. (2008) conducted a balance test after applying a commercial cold gel and cold spray. They observed a significant difference in the single-leg static balance test and only after applying a cold pack. Ingersoll et al. (1992) assessed postural balance, but none of the dependent variables changed significantly after cryotherapy. Analyzing the impact of WBC, one can only find work on patients with multiple sclerosis. The aim of the study by Staszkiewicz et al. (2025) was to evaluate the effect of 10 and 20 WBC series without kinesiotherapy on disability, assessed by muscle spasticity, and body balance in patients with multiple sclerosis. A small improvement in stability was observed in patients with multiple sclerosis following whole-body cryotherapy. The authors concluded that WBC without subsequent targeted kinesiotherapy is not sufficient to achieve clear and measurable benefits in the rehabilitation of patients with multiple sclerosis (Staszkiewicz et al., 2025).

The available literature still lacks reports on the effects of whole-body cryotherapy on biomechanical balance indices in healthy individuals (Beelen et al., 2022). Therefore, the aim of this study was to determine the effect of a series of 10 whole-body cryotherapy treatments on selected static balance indices in healthy, young women not participating in competitive sports. The analysis was based on the results of studies using the FreeMed stabilometric platform. It was hypothesized that the balance index would not change or improve after the use of WBC.

## 2 Materials and methods

### 2.1 Participant characteristics

The presented study, consistent with the assumptions of the Helsinki Declaration; approval number of the Bioethical Committee of the District Medical Chamber in Krakow: 64/KBL/OIL/2024 of 04/07/2024; each volunteer read the information about the study design and was given the opportunity to ask questions, after which he gave informed written consent to participate in the study; A physiotherapist took care of the participants’ safety. This study was designed as a prospective, single-arm, repeated-measures intervention study.

**Figure 1:**
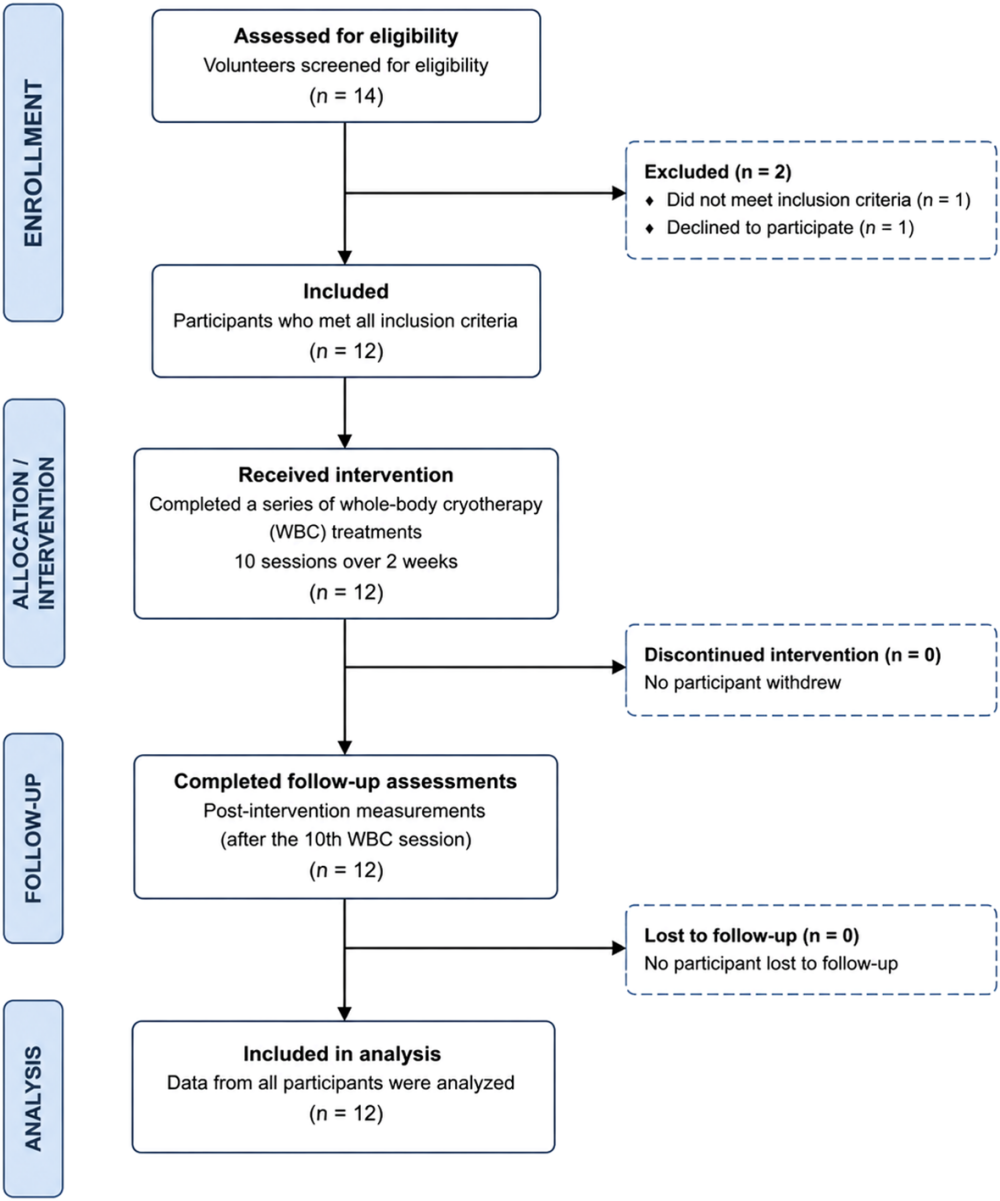
TREND flow chart. WBC, whole-body cryotherapy.

The study involved 12 (n=12) women aged 21–25 years (mean age: 22.58 ± 1.68 years). The participants had an average BMI of 20.83, with a height of 158–182 cm (mean height: 168.00 ± 0.07 cm). Measurements took place at the Academy of Physical Culture in Krakow and at the Małopolska Cryotherapy Center in Krakow. Participant recruitment took place from March to May 2025. To ensure homogeneity within the study group, the participants did not regularly engage in high-intensity physical activity (professional training). The participants were healthy (without chronic diseases) and were required to undergo medical examinations, which also ruled out contraindications to WBC treatments. Main exclusion criteria: chronic diseases, infections during the study period, pregnancy, elevated temperature, cardiovascular diseases, coagulation disorders, hypersensitivity to cold, metal implants, use of stimulants, ongoing use of medications that may affect the parameters being tested, previous lower limb or spine injury, change in physical activity immediately before and during the project, change in diet immediately before and during the project. All participants formed a cohesive group of women who led a moderately active lifestyle, engaged in similarly intense occupational activities, and were not sedentary. In all subjects, the right lower limb was the dominant lower limb. The participants followed a healthy, balanced diet (Mediterranean or DASH (Dietary Approaches to Stop Hypertension)). None of the women had previously undergone whole-body or local cryotherapy treatments. All of these guidelines allowed for the achievement of results suggesting an independent intervention, unaffected by external factors such as physical activity or health status. None of the subjects reported any changes or adverse events during the project. No participants withdrew from the study and no missing data occurred (**Figure 1**).

### 2.2 Measuring tools

**Figure 2:**
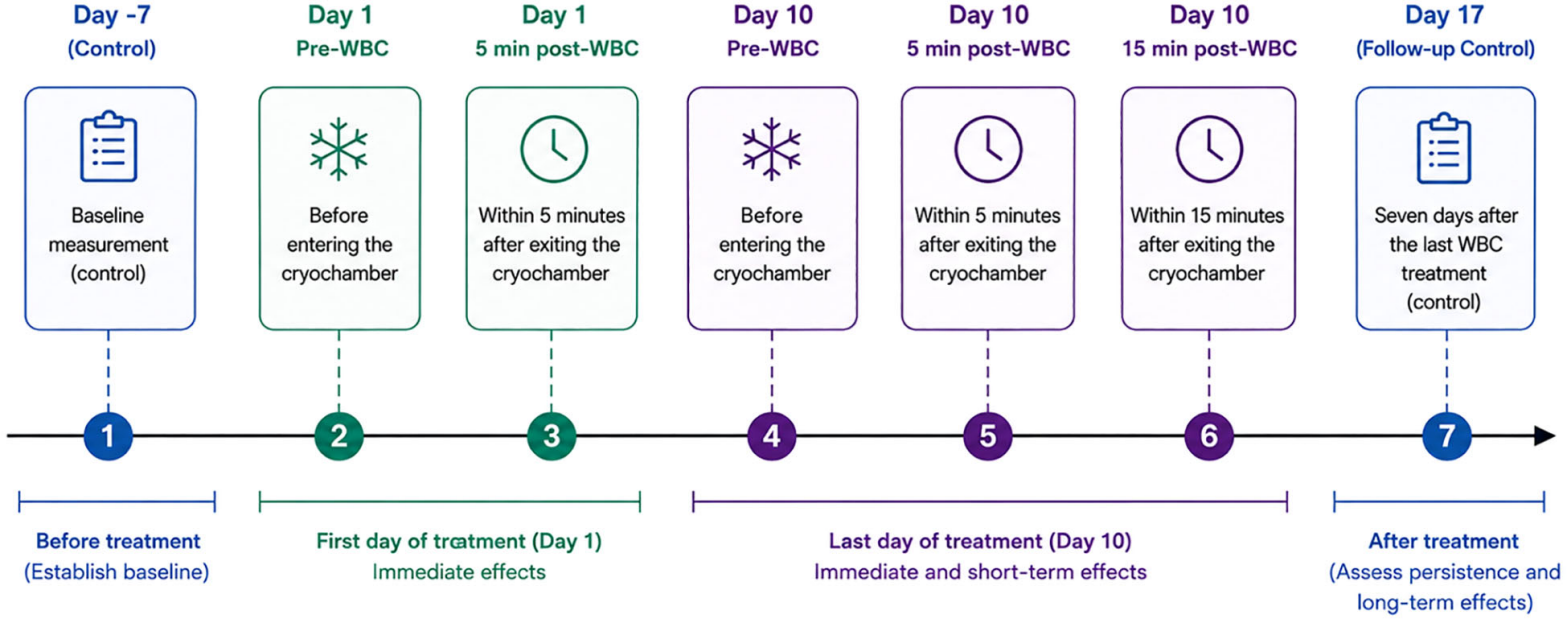
Timeline of skin assessments performed during the study. Measurements were obtained seven days before the first whole-body cryotherapy (WBC) session (control), immediately before and 5 min after the first session, immediately before and 5 and 15 min after the final (10th day) session, and seven days after completion of the treatment series (follow-up control). All assessments were conducted between 3:00 PM and 4:00 PM to minimize circadian variation.

Balance parameters were assessed seven times over a precisely defined period to capture both immediate and long-term changes. The first measurements were taken seven days before the start of the WBC treatment (control), which served to establish baseline status. Then, on the first day of the treatment series, measurements were taken twice: before entering the cryochamber and within 5 minutes of exiting. On the last day (day 10) of the treatment series, measurements were taken three times: before entering the cryochamber, 5 minutes after exiting, and 15 minutes after exiting. The final measurement (control) was taken seven days after the last WBC treatment to assess persistence and long-term effects. The tests were always performed at the same time of day, between 3:00 PM and 4:00 PM (**Figure 2**).

Static balance was assessed using stabilography, which involves the quantitative analysis of COP displacements while maintaining a standing position. The recorded postural sway reflects the continuous activity of the neuromuscular system responsible for postural stabilization, even in conditions of apparent immobility (Duarte and Freitas, 2010; Scoppa et al., 2013). Measurements were performed using the FreeMed stabilometric platform (Sensor Medica, Italy) in both bipedal and unipedal standing conditions, with eyes open (EO) and closed (EC). The use of varied sensory conditions and a reduced support area increase the load on the balance system and allow for a more accurate assessment of the mechanisms regulating postural stability in healthy individuals (Mochizuki et al., 2006). During the test, all participants’ upper limbs were relaxed alongside their torsos, with their gaze forward, and their non-supporting lower limb was flexed at the knee and hip joints (approximately 20 cm above the ground) while standing on one leg. Each participant completed one familiarization trial before their first recorded trial to ensure they understood the task and were able to maintain the required posture. The tests were always conducted under the same environmental conditions. Test duration: approximately 2 minutes Not all participants began the test on the same day, so it was possible to complete the test within a short time frame. The analyzed balance parameters are presented below.

Center of Pressure Path Length (COP Path Length).

This parameter determines the total length of the COP displacement path during the test. It is one of the most commonly used indicators of postural stability, as it reflects the overall corrective activity of the neuromuscular system. An increase in COP path length is interpreted as a decrease in balance control efficiency (Palmieri et al., 2002; Raymakers et al., 2005; Paillard and Noé, 2015). Examined:

- mean COP Velocity (Mean Velocity) - The mean velocity of sway describes the rate of stabilization reactions and the dynamics of postural adjustment. This parameter is considered particularly sensitive to changes in sensory conditions and the difficulty of the postural task. Higher values of mean COP velocity indicate greater postural instability;
- maximum COP sway in the anteroposterior and lateral axes (max AP, max ML) - These stabilometric indicators describe the extreme values of center of pressure displacement in the sagittal and frontal planes. They enable the assessment of the extent of postural control and the identification of directions in which postural stability is most challenged. These parameters are particularly sensitive to changes resulting from the elimination of visual control or a reduction in the support area;
- mean X and Y (mean Y) COP position - The mean values of the center of pressure position in the lateral and anteroposterior axes provide information on the distribution of body load and possible weight shifts within the support area. These indicators are used to assess postural symmetry and the tendency to preferentially load specific areas of the foot;
- forefoot and Hindfoot Load Distribution - Analysis of forefoot and hindfoot load allows for the assessment of how the center of mass is adjusted relative to the base of support. Changes in this area may indicate modifications in postural stabilization patterns and adaptation of the balance system to the test conditions (Winter et al., 1998; Palmieri et al., 2002; Mochizuki et al., 2006; Duarte and Freitas, 2010; Scoppa et al., 2013; Paillard and Noé, 2015).

### 2.3 Description of the intervention

Each WBC treatment was conducted in two stages. Participants initially spent approximately 20–30 seconds in a vestibule with a temperature of -60 degrees Celsius, then moved to a target chamber, where they remained for three minutes at -120 degrees Celsius. During exposure to low temperatures, participants were required to wear protective equipment, including gloves, a hat, a mask covering their mouth and nose, long cotton or wool socks, and wooden shoes. Treatments were performed daily from Monday to Friday at a fixed time (3:00-4:00 PM) for two weeks.

### 2.4 Statistical analysis

The empirical analysis allowed for the presentation of descriptive statistics for the studied parameter in the form of means, standard deviations, and minimum and maximum values. The normality of the distribution of variables was verified using the Shapiro-Wilk test. The ANOVA test for repeated measures was used to determine the significance of changes within the study group depending on the time of measurement. In the case of statistically significant differences in the analysis of variance, *post-hoc* tests (The Bonferroni test was used as a *post hoc* analysis following ANOVA to identify which group means differed significantly from each other) were performed. The effect sizes were calculated using partial η² corresponding to the statistical tests employed in the analyses. The significance level for the analyses was p=0.05. All calculations were performed using Statistica 13 software (Tibco Software Inc., USA).

## 3 Results

The average results of individual measurements are given in **Tables 1**–**3**. Statistical analysis revealed significant differences in the mean values of selected balance parameters, which were verified using repeated measures ANOVA (**Tables 1**–**4**). The analysis revealed significant:

**Table 1:** Tested indicators (mean ± standard deviation) and analysis of variance for repeated measurements of the tested indicators – F test value and p significance level - in conditions of standing on both feet.

	Parameter	I	II	III	IV	V	VI	VII	F	p	Eta-square/effect
EO	COP sway path length (mm)	455.15 ± 107.52	382.53 ± 78.68	439.53 ± 149.32	355.70 ± 87.66	389.55 ± 75.24	360.79 ± 96.90	436.35 ± 134.55	2.47	0.03	0.18 large
	mean COP displacement velocity (mm/s)	15.29 ± 3.63	12.82 ± 2.65	14.75 ± 4.99	11.92 ± 2.93	13.07 ± 2.52	12.09 ± 3.25	14.62 ± 4.52	2.50	0.03	0.18 large
	maximum COP oscillation (mm)	2.81 ± 0.92	2.95 ± 1.62	5.48 ± 9.52	3.31 ± 1.85	3.23 ± 1.46	3.25 ± 2.02	3.67 ± 1.83	0.65	0.69	0.06
	mean COP position in the X axis (mm)	-11.43 ± 4.42	-8.73 ± 12.37	-11.50 ± 8.53	-11.92 ± 6.92	-9.42 ± 9.63	-8.56 ± 13.59	-11.43 ± 13.75	0.33	0.92	0.03
	mean COP position in the Y axis (mm)	-17.52 ± 17.03	-11.13 ± 15.40	-14.49 ± 17.04	-9.70 ± 20.69	-13.42 ± 21.42	-12.12 ± 25.11	-8.29 ± 21.59	0.95	0.46	0.08
	loading of the left forefoot (%)	43.58 ± 12.12	48.17 ± 12.10	50.08 ± 11.56	50.00 ± 17.05	45.42 ± 18.58	46.50 ± 19.98	49.25 ± 19.86	1.19	0.32	0.10
	loading of the left hindfoot (%)	56.42 ± 12.12	51.83 ± 12.10	49.92 ± 11.56	51.25 ± 16.47	54.58 ± 18.58	53.50 ± 19.98	50.75 ± 19.86	1.03	0.41	0.09
	loading of the right forefoot (%)	36.42 ± 12.46	43.50 ± 12.38	42.42 ± 13.97	42.58 ± 15.70	39.25 ± 16.99	41.50 ± 16.12	46.42 ± 12.69	1.49	0.19	0.12
	loading of the right hindfoot (%)	63.58 ± 12.46	56.50 ± 12.38	57.58 ± 13.97	57.42 ± 15.70	60.75 ± 16.99	58.50 ± 16.12	53.58 ± 12.69	1.49	0.19	0.12
EC	COP sway path length (mm)	496.09 ± 140.26	416.52 ± 138.73	444.49 ± 138.25	418.33 ± 100.15	439.52 ± 87.15	412.08 ± 100.77	438.68 ± 132.24	1.39	0.23	0.11
	mean COP displacement velocity (mm/s)	16.67 ± 4.76	13.99 ± 4.74	14.95 ± 4.69	14.02 ± 3.35	14.76 ± 2.91	13.83 ± 3.38	14.72 ± 4.45	1.36	0.24	0.11
	maximum COP oscillation (mm)	3.03 ± 0.89	2.40 ± 0.64	2.23 ± 0.79	2.93 ± 1.60	2.27 ± 0.56	2.33 ± 0.78	2.59 ± 1.23	1.84	0.11	0.14
	mean COP position in the X axis (mm)	-8.22 ± 14.91	-8.32 ± 14.83	-10.90 ± 9.45	-11.61 ± 8.24	-9.41 ± 10.04	-8.03 ± 11.03	-9.24 ± 14.49	0.35	0.91	0.03
	mean COP position in the Y axis (mm)	-17.64 ± 18.23	-12.65 ± 17.70	-14.16 ± 15.85	-10.68 ± 22.38	-15.72 ± 18.96	-16.67 ± 15.97	-8.19 ± 19.59	1.68	0.14	0.13
	loading of the left forefoot (%)	43.25 ± 13.60	47.25 ± 13.03	46.00 ± 12.88	48.00 ± 16.69	42.33 ± 16.71	41.25 ± 15.17	48.75 ± 17.00	2.75	0.02	0.20 large
	loading of the left hindfoot (%)	56.75 ± 13.60	52.75 ± 13.03	54.00 ± 12.88	52.00 ± 16.69	57.67 ± 16.71	58.75 ± 15.17	51.25 ± 17.00	2.72	0.02	0.20 large
	loading of the right forefoot (%)	37.25 ± 13.79	41.92 ± 11.78	40.42 ± 13.71	41.50 ± 17.43	36.67 ± 15.49	38.25 ± 12.89	46.58 ± 11.81	2.22	0.05	0.17
	loading of the right hindfoot (%)	62.75 ± 13.79	58.08 ± 11.78	59.58 ± 13.71	58.50 ± 17.43	63.33 ± 15.49	61.75 ± 12.89	53.42 ± 11.81	2.22	0.05	0.17

EO, eyes open; EC, eyes closed.

**Table 2:** Tested indicators (mean ± standard deviation) and analysis of variance for repeated measurements of the tested indicators – F test value and p significance level - in the condition of standing on one leg on the left leg.

	Parameter	I	II	III	IV	V	VI	VII	F	p	Eta-square/Effect
EO	COP oscillation path length (mm)	269.30 ± 99.72	249.69 ± 77.62	235.88 ± 67.18	236.79 ± 85.91	225.52 ± 55.26	220.57 ± 44.91	224.54 ± 55.98	1.63	0.15	0.13
	average COP displacement velocity (mm/s)	26.95 ± 10.04	24.88 ± 7.75	23.49 ± 6.66	22.46 ± 9.78	22.49 ± 5.51	22.00 ± 4.52	22.37 ± 5.54	1.59	0.16	0.13
	maximum COP oscillation (mm)	5.84 ± 2.84	5.91 ± 1.97	5.20 ± 1.48	5.56 ± 3.44	4.83 ± 1.80	4.67 ± 1.22	4.82 ± 1.62	0.99	0.44	0.08
	average COP position in the X axis (mm)	0.06 ± 5.14	-1.22 ± 4.93	-2.45 ± 3.51	-1.69 ± 3.16	-0.76 ± 4.69	-1.88 ± 4.51	-3.94 ± 3.45	2.46	0.03	0.18 large
	average COP position in the Y axis (mm)	-17.90 ± 12.20	-12.74 ± 7.34	-11.59 ± 10.97	-10.75 ± 13.14	-17.95 ± 10.84	-13.75 ± 11.79	-7.92 ± 12.97	4.42	0.00	0.29 large
EC	COP oscillation path length (mm)	708.48 ± 217.52	658.95 ± 205.95	631.46 ± 256.72	595.49 ± 231.87	547.45 ± 197.59	544.40 ± 212.18	540.40 ± 118.80	2.35	0.04	0.18 large
	average COP displacement velocity (mm/s)	70.63 ± 21.70	65.76 ± 20.60	63.08 ± 25.76	59.35 ± 23.23	54.57 ± 19.73	54.33 ± 21.27	53.93 ± 11.88	2.33	0.04	0.18 large
	maximum COP oscillation (mm)	16.99 ± 5.67	16.31 ± 6.09	13.80 ± 6.52	12.40 ± 5.21	12.78 ± 5.23	11.53 ± 5.20	11.60 ± 3.41	0.62	0.72	0.05
	average COP position in the X axis (mm)	-0.39 ± 5.22	-1.13 ± 6.25	-3.14 ± 5.99	-0.57 ± 4.77	-1.00 ± 6.64	-1.22 ± 6.37	-2.24 ± 3.29	2.02	0.08	0.16
	average COP position in the Y axis (mm)	-23.25 ± 21.82	-18.08 ± 11.12	-15.09 ± 15.33	-13.16 ± 14.09	-18.52 ± 12.25	-16.65 ± 14.33	-13.90 ± 16.13	4.52	0.00	0.29 large

EO, eyes open; EC, eyes closed.

**Table 3:** Tested indicators (mean ± standard deviation) and analysis of variance for repeated measurements of the tested indicators – F test value and p significance level - in the condition of standing on one leg on the right leg.

Parameter		I	II	III	IV	V	VI	VII	F	p	Eta-square/Effect
EO	COP oscillation path length (mm)	285.45 ± 54.71	302.73 ± 77.95	308.76 ± 88.44	300.26 ± 104.90	269.90 ± 113.95	271.99 ± 105.39	261.56 ± 67.32	1.10	0.37	0.09
	average COP displacement velocity (mm/s)	28.47 ± 5.45	28.67 ± 6.90	30.75 ± 8.80	29.88 ± 10.45	26.89 ± 11.36	27.13 ± 10.48	26.08 ± 6.69	0.88	0.51	0.07
	maximum COP oscillation (mm)	6.41 ± 2.73	6.06 ± 1.88	7.34 ± 2.62	10.27 ± 13.69	6.19 ± 3.02	6.13 ± 2.90	5.78 ± 2.08	0.95	0.47	0.08
	average COP position in the X axis (mm)	-1.00 ± 5.32	2.52 ± 4.92	0.09 ± 6.78	6.10 ± 18.11	3.88 ± 8.66	3.15 ± 7.33	3.60 ± 7.70	1.32	0.26	0.11
	average COP position in the Y axis (mm)	-19.44 ± 8.41	-16.16 ± 9.03	-17.50 ± 11.21	-13.48 ± 10.93	-18.77 ± 9.98	-17.62 ± 14.32	-14.44 ± 11.85	1.50	0.19	0.12
EC	COP oscillation path length (mm)	700.64 ± 312.19	595.13 ± 215.14	578.30 ± 259.90	648.12 ± 263.68	584.60 ± 280.39	535.61 ± 277.00	554.50 ± 188.64	1.32	0.26	0.11
	average COP displacement velocity (mm/s)	69.78 ± 31.06	59.19 ± 21.51	57.83 ± 26.02	64.77 ± 26.32	58.49 ± 28.05	53.58 ± 27.75	55.28 ± 18.78	1.29	0.27	0.11
	maximum COP oscillation (mm)	16.38 ± 8.24	12.68 ± 5.11	12.44 ± 6.87	14.41 ± 6.62	13.41 ± 6.32	11.58 ± 6.05	11.54 ± 5.08	1.78	0.12	0.14
	average COP position in the X axis (mm)	-0.45 ± 6.43	-1.55 ± 6.36	-0.10 ± 7.51	-1.58 ± 4.42	1.77 ± 6.28	3.97 ± 8.02	0.21 ± 7.05	2.82	0.02	0.20 large
	average COP position in the Y axis (mm)	-20.78 ± 14.34	-22.83 ± 16.54	-20.50 ± 11.53	-21.88 ± 8.39	-23.22 ± 8.73	-16.09 ± 15.45	-16.60 ± 13.10	1.36	0.25	0.11

EO, eyes open; EC, eyes closed.

**Table 4:** Differences in means, 95% confidence intervals and *post hoc* test values for pairwise comparisons of the studied indicators.

Parameter	Measurement	Measurement	Mean difference	-95% CI	+95% CI	p
COP sway path length (mm) BF EO	I	II	72.61	0.03	145.20	0.04*
		III	15.62	-56.95	88.20	0.66
		IV	99.44	26.86	172.03	0.00*
		V	65.60	-6.97	138.18	0.07
		VI	94.36	21.78	166.94	0.01*
		VII	18.80	-53.77	91.38	0.60
	II	III	-56.99	-129.57	15.59	0.12
		IV	26.83	-45.75	99.41	0.46
		V	-7.01	-79.59	65.57	0.84
		VI	21.74	-50.83	94.32	0.55
		VII	-53.81	-126.39	18.77	0.14
	III	IV	83.82	11.24	156.40	0.02*
		V	49.98	-22.60	122.56	0.17
		VI	78.73	6.15	151.32	0.03*
		VII	3.18	-69.40	75.76	0.93
	IV	V	-33.84	-106.42	38.74	0.35
		VI	-5.08	-77.66	67.49	0.88
		VII	-80.64	-153.22	-8.05	0.02*
	V	VI	28.75	-43.82	101.34	0.43
		VII	-46.80	-119.38	25.78	0.20
	VI	VII	-75.55	-148.14	-2.97	0.04*
mean COP displacement velocity (mm/s) BF EO	I	II	2.47	0.03	4.90	0.04*
		III	0.54	-1.89	2.97	0.65
		IV	3.36	0.93	5.80	0.00*
		V	2.21	-0.22	4.64	0.07
		VI	3.19	0.76	5.63	0.01*
		VII	0.66	-1.77	3.09	0.58
	II	III	-1.93	-4.36	0.50	0.11
		IV	0.89	-1.53	3.33	0.46
		V	-0.25	-2.69	2.17	0.83
		VI	0.72	-1.70	3.16	0.55
		VII	-1.80	-4.24	0.62	0.14
	III	IV	2.82	0.39	5.26	0.02*
		V	1.67	-0.76	4.10	0.17
		VI	2.65	0.22	5.09	0.03*
		VII	0.12	-2.31	2.55	0.92
	IV	V	-1.15	-3.59	1.27	0.34
		VI	-0.17	-2.60	2.26	0.88
		VII	-2.70	-5.14	-0.27	0.02*
	V	VI	0.98	-1.45	3.42	0.42
		VII	-1.55	-3.98	0.88	0.20
	VI	VII	-2.53	-4.97	-0.09	0.04*
loading of the left forefoot (%) BF EC	I	II	-4.00	-9.08	1.08	0.12
		III	-2.75	-7.83	2.33	0.28
		IV	-4.75	-9.83	0.33	0.06
		V	0.91	-4.16	5.99	0.71
		VI	2.00	-3.08	7.08	0.43
		VII	-5.50	-10.58	-0.41	0.03*
	II	III	1.25	-3.83	6.33	0.62
		IV	-0.75	-5.83	4.33	0.76
		V	4.91	-0.16	9.99	0.05
		VI	6.00	0.91	11.08	0.02*
		VII	-1.50	-6.58	3.58	0.55
	III	IV	-2.00	-7.08	3.08	0.43
		V	3.66	-1.41	8.74	0.15
		VI	4.75	-0.33	9.83	0.06
		VII	-2.75	-7.83	2.33	0.28
	IV	V	5.66	0.58	10.74	0.02*
		VI	6.75	1.66	11.83	0.01*
		VII	-0.75	-5.83	4.33	0.76
	V	VI	1.08	-3.99	6.16	0.67
		VII	-6.41	-11.49	-1.33	0.01*
	VI	VII	-7.50	-12.58	-2.41	0.00*
loading of the left hindfoot (%) BF EC	I	II	4.00	-1.08	9.08	0.12
		III	2.75	-2.33	7.83	0.28
		IV	4.75	-0.33	9.83	0.06
		V	-0.91	-5.99	4.16	0.71
		VI	-2.00	-7.08	3.08	0.43
		VII	5.50	0.41	10.58	0.03*
	II	III	-1.25	-6.33	3.83	0.62
		IV	0.75	-4.33	5.83	0.76
		V	-4.91	-9.99	0.16	0.05
		VI	-6.00	-11.08	-0.91	0.02*
		VII	1.50	-3.58	6.58	0.55
	III	IV	2.00	-3.08	7.08	0.43
		V	-3.66	-8.74	1.41	0.15
		VI	-4.75	-9.83	0.33	0.06
		VII	2.75	-2.33	7.83	0.28
	IV	V	-5.66	-10.74	-0.58	0.02*
		VI	-6.75	-11.83	-1.66	0.01*
		VII	0.75	-4.33	5.83	0.76
	V	VI	-1.08	-6.16	3.99	0.67
		VII	6.41	1.33	11.49	0.01*
	VI	VII	7.50	2.41	12.58	0.00*
average COP position in the X axis (mm) LL EO	I	II	1.27	-1.03	3.57	0.27
		III	2.50	0.19	4.81	0.03*
		IV	1.74	-0.55	4.05	0.13
		V	0.81	-1.49	3.11	0.48
		VI	1.93	-0.37	4.23	0.09
		VII	3.99	1.69	6.30	0.00*
	II	III	1.23	-1.07	3.53	0.28
		IV	0.47	-1.82	2.78	0.68
		V	-0.46	-2.76	1.84	0.69
		VI	0.65	-1.64	2.96	0.57
		VII	2.72	0.41	5.03	0.02*
	III	IV	-0.75	-3.06	1.54	0.51
		V	-1.69	-3.99	0.61	0.14
		VI	-0.57	-2.87	1.73	0.62
		VII	1.49	-0.81	3.79	0.20
	IV	V	-0.93	-3.24	1.36	0.41
		VI	0.18	-2.12	2.48	0.87
		VII	2.24	-0.05	4.55	0.05
	V	VI	1.11	-1.18	3.42	0.33
		VII	3.18	0.88	5.49	0.00*
	VI	VII	2.06	-0.23	4.37	0.07
average COP position in the Y axis (mm) LL EO	I	II	-5.16	-10.12	-0.20	0.04*
		III	-6.31	-11.27	-1.35	0.01*
		IV	-7.14	-12.10	-2.18	0.00*
		V	0.05	-4.90	5.01	0.98
		VI	-4.15	-9.10	0.80	0.09
		VII	-9.98	-14.94	-5.02	0.00*
	II	III	-1.14	-6.10	3.80	0.64
		IV	-1.98	-6.93	2.97	0.42
		V	5.21	0.26	10.17	0.03*
		VI	1.01	-3.94	5.97	0.68
		VII	-4.82	-9.77	0.13	0.05
	III	IV	-0.83	-5.79	4.12	0.73
		V	6.36	1.40	11.32	0.01*
		VI	2.16	-2.79	7.12	0.38
		VII	-3.67	-8.62	1.28	0.14
	IV	V	7.20	2.24	12.15	0.00*
		VI	2.99	-1.96	7.95	0.23
		VII	-2.83	-7.79	2.11	0.25
	V	VI	-4.20	-9.16	0.75	0.09
		VII	-10.03	-14.99	-5.08	0.00*
	VI	VII	-5.83	-10.79	-0.87	0.02*
COP oscillation path length (mm) LL EC	I	II	49.52	-70.70	169.76	0.41
		III	77.01	-43.21	197.25	0.20
		IV	112.98	-7.25	233.22	0.06
		V	161.02	40.79	281.26	0.00*
		VI	164.07	43.83	284.30	0.00*
		VII	168.07	47.84	288.31	0.00*
	II	III	27.49	-92.74	147.72	0.64
		IV	63.45	-56.77	183.69	0.29
		V	111.49	-8.73	231.73	0.06
		VI	114.54	-5.68	234.78	0.06
		VII	118.55	-1.68	238.78	0.05
	III	IV	35.96	-84.26	156.20	0.55
		V	84.00	-36.22	204.24	0.16
		VI	87.05	-33.17	207.29	0.15
		VII	91.06	-29.17	211.29	0.13
	IV	V	48.04	-72.19	168.27	0.42
		VI	51.08	-69.14	171.32	0.39
		VII	55.09	-65.14	175.32	0.36
	V	VI	3.04	-117.18	123.28	0.95
		VII	7.05	-113.18	127.28	0.90
	VI	VII	4.00	-116.23	124.23	0.94
average COP displacement velocity (mm/s) LL EC	I	II	4.87	-7.15	16.91	0.42
		III	7.55	-4.47	19.59	0.21
		IV	11.28	-0.75	23.31	0.06
		V	16.06	4.03	28.09	0.00*
		VI	16.30	4.27	28.34	0.00*
		VII	16.70	4.67	28.74	0.00*
	II	III	2.68	-9.35	14.71	0.65
		IV	6.40	-5.63	18.43	0.29
		V	11.18	-0.84	23.22	0.06
		VI	11.43	-0.60	23.46	0.06
		VII	11.83	-0.20	23.86	0.05
	III	IV	3.72	-8.31	15.75	0.53
		V	8.50	-3.52	20.53	0.16
		VI	8.75	-3.28	20.78	0.15
		VII	9.15	-2.88	21.18	0.13
	IV	V	4.78	-7.24	16.81	0.43
		VI	5.02	-7.00	17.06	0.40
		VII	5.42	-6.60	17.46	0.37
	V	VI	0.24	-11.79	12.27	0.96
		VII	0.64	-11.39	12.67	0.91
	VI	VII	0.40	-11.63	12.43	0.94
maximum COP oscillation (mm) LL EC	I	II	0.68	-2.25	3.61	0.64
		III	3.19	0.25	6.12	0.03*
		IV	4.58	1.65	7.52	0.00*
		V	4.20	1.27	7.14	0.00*
		VI	5.45	2.52	8.39	0.00*
		VII	5.39	2.46	8.32	0.00*
	II	III	2.51	-0.42	5.44	0.09
		IV	3.90	0.97	6.83	0.00*
		V	3.52	0.59	6.45	0.01*
		VI	4.77	1.84	7.71	0.00*
		VII	4.71	1.77	7.64	0.00*
	III	IV	1.39	-1.53	4.32	0.34
		V	1.01	-1.91	3.94	0.49
		VI	2.26	-0.66	5.19	0.12
		VII	2.20	-0.73	5.13	0.13
	IV	V	-0.38	-3.31	2.55	0.79
		VI	0.87	-2.06	3.80	0.55
		VII	0.80	-2.12	3.73	0.58
	V	VI	1.25	-1.68	4.18	0.39
		VII	1.18	-1.74	4.11	0.42
	VI	VII	-0.06	-2.99	2.86	0.96
average COP position in the X axis (mm) RL EC	I	II	1.10	-2.21	4.41	0.50
		III	-0.34	-3.65	2.97	0.83
		IV	1.12	-2.18	4.44	0.49
		V	-2.21	-5.53	1.09	0.18
		VI	-4.41	-7.72	-1.09	0.00*
		VII	-0.65	-3.96	2.66	0.69
	II	III	-1.44	-4.75	1.86	0.38
		IV	0.02	-3.28	3.34	0.98
		V	-3.32	-6.63	-0.00	0.04*
		VI	-5.51	-8.83	-2.20	0.00*
		VII	-1.75	-5.06	1.55	0.29
	III	IV	1.47	-1.84	4.78	0.37
		V	-1.87	-5.19	1.43	0.26
		VI	-4.07	-7.38	-0.75	0.01*
		VII	-0.31	-3.62	3.00	0.85
	IV	V	-3.34	-6.66	-0.03	0.04*
		VI	-5.54	-8.85	-2.22	0.00*
		VII	-1.78	-5.09	1.53	0.28
	V	VI	-2.19	-5.50	1.12	0.19
		VII	1.56	-1.74	4.88	0.34
	VI	VII	3.76	0.44	7.07	0.02*

EO, eyes open; EC, eyes closed; BF, both foot; LL, left leg; RL, right leg; *statistically significant.

- decrease in the COP sway path length during bipedal stance with EO, with the largest difference observed between measurement I and IV and III and IV. Significant decreases also occurred between measurement I and VI and III and VI (F = 2.47, p = 0.03);
- decrease in the average COP displacement velocity during bipedal stance with EO, with the largest difference observed between measurement I and IV and III and IV. Significant decreases also occurred between measurement I and VI and III and VI (F = 2.50, p = 0.03);
- decrease in the load on the forefoot of the left foot in the condition of standing with both feet with EC, with the greatest difference noted between measurement II and VI and between measurement IV and VI (F = 2.75, p=0.02);
- decrease in the load on the left hindfoot during double-leg stance with EC, with the largest difference noted between measurement II and VI and IV and VI (F = 2.72, p = 0.02);
- decrease in the mean COP position in the X-axis during single-leg stance on the left foot with EO, with the largest difference noted between measurement I and VII (F = 2.46, p = 0.03);
- decrease in the mean COP position in the Y-axis during single-leg stance on the left foot with EO, with the largest difference noted between measurement I and VII; a significant decrease also occurred between measurement V and VII (F = 4.42, p = 0.00);
- decrease in the COP sway path length during single-legged standing on the left foot with EC, with the greatest difference observed between measurement I and VII. Significant decreases also occurred between measurement I and VI, I and V (F = 2.35, p=0.04);
- decrease in the average COP displacement velocity during single-legged standing on the left foot with EC, with the greatest difference observed between measurement I and VII. Significant decreases also occurred between measurement I and VI, I and V (F = 2.33, p=0.04);
- decrease in the maximum COP oscillations during single-legged standing on the left foot with EC, with the greatest difference observed between measurement I and VI. Significant decreases also occurred between measurement I and VII, I and IV (F = 4.52, p=0.00);
- increase in the COP sway path length during bipedal stance with EO, with the greatest difference observed between measurement IV and VII, and a significant increase between measurement IV and VI (F = 2.47, p = 0.03);
- increase in the mean COP displacement velocity during bipedal stance with EO, with the greatest difference observed between measurement IV and VII, and a significant increase between measurement IV and VI (F = 2.50, p = 0.03);
- increase in the maximum COP oscillations during unipedal stance on the left foot with EC, with the greatest difference observed between measurement III and I, and a significant increase between measurement III and II (F = 4.52, p = 0.00);
- increase in this indicator indicates a change in postural control strategy. an increase in the mean COP position in the X axis during single-legged stance on the right foot with EC, with the greatest difference noted between measurement VI and II; a significant increase was also observed between measurement VI and IV (F = 2.82, p=0.02).

## 4 Discussion

The aim of this study was to assess the effect of a series of WBC treatments on selected parameters of static balance in healthy young women. Analysis of the obtained results indicates that exposure to very low temperatures could induce measurable changes in global indicators of postural stability, such as the COP sway path length and the mean COP displacement velocity, as well as in selected parameters describing the distribution of foot load and the position of the center of pressure in the anteroposterior and lateral axes. The nature of the observed changes suggests an adaptive process involving reorganization of neuromuscular control and modulation of proprioceptive information processing.

In bipedal stance with EO, a significant decrease in the COP sway path length was observed between study I (455.15 mm) and study IV (355.70 mm), representing a reduction of approximately 22%. A similar trend was observed in the mean COP displacement velocity, where a decrease from 15.29 mm/s to 11.92 mm/s was observed. The COP velocity is an indicator of the participant’s postural control and characterizes the net neuromuscular activity required to maintain balance (Duarte and Freitas, 2010) and is a common parameter used in scientific research (Julienne et al., 2024). It is worth emphasizing that the literature indicates that changes in COP path length exceeding 10-15% can be interpreted as exceeding natural measurement variability. In the present study, this reduction is significantly greater, strengthening the argument that the observed effect is not solely the result of task habituation or learning effects. More recent studies also confirm that COP path length remains one of the most reliable parameters for assessing subtle balance changes in young and healthy individuals (Santos et al., 2010). Reductions in COP path length and velocity indicate more economical postural regulation, which can be interpreted as improved efficiency of neuromuscular control.

From a biomechanical perspective, maintaining a standing position on a stable surface relies primarily on a jumping strategy, in which control is achieved through precise modulation of ankle muscle tension (Winter, 1995). Reductions in global sway may therefore indicate stabilization of calf muscle activity and more precise regulation of the moment of force at this joint. However, subsequent measurements revealed a partial increase in COP length and velocity. This biphasic pattern of changes—initial improvement, followed by partial stabilization or regression—may reflect a neuroadaptation mechanism. Maurer and Peterka (2005) suggest that spontaneous COP fluctuations result from a dynamic reorganization of feedback mechanisms between the sensory and motor systems (Maurer and Peterka, 2005). The initial reduction in sway may be associated with increased postural stiffness, while the subsequent increase may be associated with a return to more flexible, adaptive control.

A more pronounced and unidirectional effect was observed under conditions of visual control deprivation. During single-legged stance with EC, particularly on the left limb, a systematic decrease in COP path length, mean velocity, and maximum oscillations was observed. Comparison of measurement conditions indicates that the effect of cryotherapy was stronger in tasks requiring greater involvement of the proprioceptive system. This may suggest that cryotherapy primarily affects compensatory mechanisms activated in situations of limited visual information (Peterka, 2002). Improved stability in such conditions may therefore indicate improved somatosensory integration. Contemporary models of postural control emphasize that sensory integration is a plastic process susceptible to modulation even in young individuals (Forbes et al., 2018). Neurophysiological studies conducted in recent years demonstrate that postural control engages not only core and subcortical structures but also cortical areas responsible for sensorimotor integration (Hwang et al., 2016). It is therefore possible that a series of cryotherapy treatments influenced the central mechanisms of muscle tone regulation and postural coordination.

The analysis of the results highlights the fact that the changes were more pronounced in the left limb. This may be due to the functional dominance of the lower limb or differences in motor control between the two sides of the body. The literature confirms that asymmetries in postural control can be present in healthy individuals (Forbes et al., 2018). It is therefore possible that cryotherapy had a stronger effect on the side with initially lower stability, which translated into more pronounced changes in COP parameters.

In terms of nervous system function, exposure to low temperature is associated with changes in nerve conduction and the activity of proprioceptive receptors. Algafly and George (2007) demonstrated that cryotherapy leads to a decrease in conduction velocity in sensory and motor fibers (Algafly and George, 2007). More recent literature reviews indicate that the effect of cold on proprioception and motor control depends on the duration and number of exposures (Bleakley et al., 2012). Therefore, it is possible that the observed improvement in parameters in the final measurements is the result of gradual adaptation of the neuromuscular system to the repeated cold stimulus. Furthermore, a series of WBC treatments has been shown to influence the body’s autonomic regulation by increasing parasympathetic activity (Lombardi et al., 2017). Changes in autonomic regulation may indirectly influence muscle tone and postural stability.

A reduction in the maximum COP oscillations during single-legged stance under OZ suggests greater stability in situations requiring intensive postural corrections. Horak (2006) indicates that balance control relies on the flexible use of various postural strategies (Horak, 2006). Reductions in maximum oscillations may therefore indicate stabilization of regulatory mechanisms.

Significant changes in the load distribution between the forefoot and hindfoot of the left foot under OZ conditions suggest a modification of the biomechanical balance strategy. The distribution of ground reaction forces reflects how the body controls the position of the center of mass relative to the base of support. Collins and De Luca (1993) indicate that changes in load distribution may reflect a transition between open-loop and closed-loop control (Collins and De Luca, 1993). In this study, a trend toward a more central COP position was observed, which may indicate improved functional stability.

From the perspective of more recent research on the reliability of stabilometry, it should be emphasized that even relatively small changes in the COP path length can have clinical significance – this review included studies involving healthy individuals as well as patients with non-specific low back pain (Ruhe et al., 2010). In the context of a young, healthy population, the observed changes may therefore reflect a real modulation of postural control, not merely random measurement variability.

It is also worth noting that research on WBC in recent years has increasingly indicated its impact on neuromuscular function and recovery, not only in the context of sports but also motor control (Bouzigon et al., 2016). The results of this study contribute to this research trend, suggesting that cryostimulation can affect balance mechanisms even in individuals without clinical deficits.

In summary, a series of WBC treatments affected selected parameters of static balance, leading to an initial reduction in global COP sway and improved stability under conditions of increased sensory load. The observed changes are adaptive in nature and suggest the possible impact of cryostimulation on sensory integration, muscle tone regulation, and central postural control mechanisms. These results indicate that WBC may influence the balance system in healthy individuals, which is an interesting direction for further research.

With regard to the issues discussed in the introduction, the obtained results should be compared to previous studies examining both the impact of WBC on the functioning of the neuromuscular system and studies examining changes in stabilometric parameters following other interventions. The available literature indicates that WBC primarily has a beneficial effect on regenerative processes, reduced muscle fatigue, and modulation of the inflammatory response. Rose et al. (2017) emphasized that exposure to very low temperatures may contribute to improved muscle function after exercise, but they did not directly analyze stabilometric parameters such as COP path length or center of pressure displacement velocity (Rose et al., 2017).

Subsequent studies indicated that cryostimulation may influence autonomic nervous system activity and muscle tone, potentially translating into changes in postural control (Lombardi et al., 2017). WBC triggers a response in the autonomic nervous system, leading to increased parasympathetic activity and increased norepinephrine concentration. Hausswirth et al. (2013) demonstrated that exposure to very low temperatures enhances parasympathetic activity, which promotes regeneration and reduces physiological stress (Hausswirth et al., 2013). Simultaneously, increased norepinephrine secretion influences the regulation of vascular tone, thermoregulatory processes, and modulation of neuromuscular excitability. These changes can lead to altered muscle tone and improved muscle coordination. One of the most important physiological effects of WBC is a reduction in nerve conduction and a reduction in pain receptor activity, which contributes to the reduction of pain and muscle spasm. Cryotherapy is commonly used to alleviate pain, swelling, and muscle spasm (Bettoni et al., 2013; Jastrząbek et al., 2013). Improved muscle oxygenation resulting from the subsequent increase in blood flow following a period of vasoconstriction may additionally support muscle regeneration and function (Hornery et al., 2005). Studies by Zalewski et al. (2014) also demonstrated a reduction in heart rate and an increase in stroke volume following cryostimulation, suggesting a reduced circulatory system burden and improved exercise economy. These mechanisms may indirectly influence the ability of muscles to precisely generate the tension necessary to maintain postural stability (Hornery et al., 2005; Zalewski et al., 2014; Lombardi et al., 2017). In our study, a reduction in COP path length and a decrease in mean displacement velocity were observed, particularly during single-legged stance with EC. This may suggest that a series of cryotherapy treatments influences the mechanisms of sensory-motor integration, especially in situations of increased proprioceptive load.

Studies on balance training and proprioceptive exercises have demonstrated a clear reduction in the COP path length and improved postural stability (Lesinski et al., 2015). Taube et al. (2008) indicated that the improvement in stabilometric parameters after training results from adaptation at the central level and improved processing of sensory information. A similar pattern of changes was observed in this study, even though the intervention used was not training-based (Taube et al., 2008). This may indicate that WBC produces an effect functionally similar to the adaptations observed after sensorimotor programs.

Furthermore, articles on repeated cold exposure have noted that the therapeutic effect may increase with subsequent treatments (Bouzigon et al., 2016). The nature of the changes observed in the submitted text, including larger differences between initial and final measurements, may confirm the presence of an adaptive process within postural control. Unlike previous studies focusing on regenerative or functional parameters, the present study includes a detailed stabilometric analysis under conditions that differentiate visual and proprioceptive control. The obtained results indicate that WBC can positively influence selected static balance parameters in healthy young women engaged in recreational physical activity, which significantly expands existing knowledge.

## 5 Conclusions

The use of a series of WBC treatments was associated with changes in selected stabilometric parameters, particularly under conditions of increased proprioceptive load. The results suggest a possible effect of WBC on postural control mechanisms in young and healthy people, but these require confirmation in randomized, controlled trials.

## 6 Limitations and future directions

When interpreting these data, it is important to consider the methodological limitations of this study: the small sample size; the lack of monitoring of fatigue, sleep quality, fluid intake; and the lack of a separate control group. To reduce measurement bias, all assessments were performed by the same investigator, at the same time of day, under standardized environmental conditions. Participants maintained their usual diet and physical activity throughout the study. Blinding was not feasible because all participants underwent the same intervention and the investigator performing the assessments was aware of the study phase. Due to the large number of analyzed stabilometric variables and the relatively small sample size, some statistically significant findings should be interpreted cautiously because of the increased risk of type I error. No *a priori* sample size calculation was performed because the study was exploratory and pilot in nature. Although the observed reduction in COP path length and velocity may indicate more efficient postural regulation, the underlying mechanisms remain speculative. The present study did not include neurophysiological or electromyographic assessments; therefore, any assumptions regarding central sensorimotor adaptation or changes in proprioceptive processing should be interpreted cautiously. Future studies should include larger samples, randomized controlled designs, and objective measures of neuromuscular function, such as surface electromyography, heart rate variability, or proprioceptive assessments.

## Data Availability

The original contributions presented in the study are included in the article/supplementary material. Further inquiries can be directed to the corresponding author.

## References

[B1] Algafly A. A. George K. P. (2007). The effect of cryotherapy on nerve conduction velocity, pain threshold and pain tolerance. Br. J. Sports Med. 41, 365–369. doi: 10.1136/bjsm.2006.031237 17224445 PMC2465313

[B2] Beelen P. E. van Dieën J. H. Prins M. R. Nolte P. A. Kingma I. (2022). The effect of cryotherapy on postural stabilization assessed by standardized horizontal perturbations of a movable platform. Gait Posture 94, 32–38. doi: 10.1016/j.gaitpost.2022.02.022 35231819

[B3] Bettoni L. Bonomi F. G. Zani V. Manisco L. Indelicato A. Lanteri P. . (2013). Effects of 15 consecutive cryotherapy sessions on the clinical output of fibromyalgic patients. Clin. Rheumatol. 32, 1337–1345. doi: 10.1007/s10067-013-2280-9 23636794

[B4] Bleakley C. M. Costello J. T. Glasgow P. D. (2012). Should athletes return to sport after applying ice? Sports Med. 42, 69–87. doi: 10.2165/11595970-000000000-00000 22121908

[B5] Bouzigon R. Grappe F. Ravier G. Dugué B. (2016). Whole- and partial-body cryostimulation/cryotherapy: Current technologies and practical applications. J. Therm Biol. 61, 67–81. doi: 10.1016/j.jtherbio.2016.08.009 27712663

[B6] Collins J. J. De Luca C. J. (1993). Open-loop and closed-loop control of posture: A random-walk analysis of center-of-pressure trajectories. Exp. Brain Res. 95, 308–318. doi: 10.1007/bf00229788 8224055

[B7] Dewhurst S. Riches P. E. De Vito G. (2007). Moderate alterations in lower limbs muscle temperature do not affect postural stability during quiet standing in both young and older women. J. Electromyogr Kinesiol 17, 292–298. doi: 10.1016/j.jelekin.2006.03.002 16698285

[B8] Douglas M. Bivens S. Pesterfield J. Clemson N. Castle W. Sole G. . (2013). Immediate effects of cryotherapy on static and dynamic balance. Int. J. Sports Phys. Ther. 8, 9–14. 23439672 PMC3578429

[B9] Duarte M. Freitas S. M. (2010). Revision of posturography based on force plate for balance evaluation. Rev. Bras. Fisioterapia (Sao Carlos (Sao Paulo Brazil)) 14, 183–192. 20730361

[B10] Forbes P. A. Chen A. Blouin J. S. (2018). Sensorimotor control of standing balance. Handb. Clin. Neurol. 159, 61–83. doi: 10.1016/B978-0-444-63916-5.00004-5 30482333

[B11] Hausswirth C. Schaal K. Le Meur Y. Bieuzen F. Filliard J. R. Volondat M. . (2013). Parasympathetic activity and blood catecholamine responses following a single partial-body cryostimulation and a whole-body cryostimulation. PloS One 8, e72658. doi: 10.1371/journal.pone.0072658 23991134 PMC3749989

[B12] Horak F. B. (2006). Postural orientation and equilibrium: What do we need to know about neural control of balance to prevent falls? Age Ageing 35 (Suppl 2), ii7–ii11. doi: 10.1093/ageing/afl077 16926210

[B13] Hornery D. J. Papalia S. Mujika I. Hahn A. (2005). Physiological and performance benefits of halftime cooling. J. Sci. Med. Sport 8, 15–25. doi: 10.1016/s1440-2440(05)80020-9 15887897

[B14] Hwang S. Agada P. Kiemel T. Jeka J. J. (2016). Identification of the unstable human postural control system. Front. Syst. Neurosci. 10, 22. doi: 10.3389/fnsys.2016.00022 27013990 PMC4786559

[B15] Ingersoll C. D. Knight K. L. Merrick M. A. (1992). Sensory perception of the foot and ankle following therapeutic applications of heat and cold. J. Athletic Training 27, 231–233. doi: 10.1249/00005768-199205001-00472 PMC1317251 16558166

[B16] Jastrząbek R. Straburzyńska-Lupa A. Rutkowski R. Romanowski W. (2013). Effects of different local cryotherapies on systemic levels of TNF-α, IL-6, and clinical parameters in active rheumatoid arthritis. Rheumatol. Int. 33, 2053–2060. doi: 10.1007/s00296-013-2692-5 23397259

[B17] Julienne A. Verbecque E. Besnard S. (2024). Normative data for instrumented posturography: A systematic review and meta-analysis. Front. Hum. Neurosci. 18, 1498107. doi: 10.3389/fnhum.2024.1498107 39743990 PMC11688309

[B18] Kim K. Suzuki K. Peake J. Ahn N. Ogawa K. Hong C. . (2014). Physiological and leukocyte subset responses to exercise and cold exposure in cold-acclimatized skaters. Biol. Sport 31, 39–48. doi: 10.5604/20831862.1086731 24917688 PMC3994584

[B19] Lesinski M. Hortobágyi T. Muehlbauer T. Gollhofer A. Granacher U. (2015). Effects of balance training on balance performance in healthy older adults: A systematic review and meta-analysis. Sports Med. 45, 1721–1738. doi: 10.1007/s40279-015-0375-y 26325622 PMC4656699

[B20] Lombardi G. Ziemann E. Banfi G. (2017). Whole-body cryotherapy in athletes: From therapy to stimulation. An updated review of the literature. Front. Physiol. 8, 258. doi: 10.3389/fphys.2017.00258 28512432 PMC5411446

[B21] Maurer C. Peterka R. J. (2005). A new interpretation of spontaneous sway measures based on a simple model of human postural control. J. Neurophysiol. 93, 189–200. doi: 10.1152/jn.00221.2004 15331614

[B22] Mochizuki L. Duarte M. Amadio A. C. Zatsiorsky V. M. Latash M. L. (2006). Changes in postural sway and its fractions in conditions of postural instability. J. Appl. Biomech 22, 51–60. doi: 10.1123/jab.22.1.51 16760567

[B23] Paillard T. Noé F. (2015). Techniques and methods for testing the postural function in healthy and pathological subjects. BioMed. Res. Int. 2015, 891390. doi: 10.1155/2015/891390 26640800 PMC4659957

[B24] Palmieri R. M. Ingersoll C. D. Stone M. B. Krause B. A. (2002). Center-of-pressure parameters used in the assessment of postural control. J. Sport Rehabil. 11, 51–66. doi: 10.1123/jsr.11.1.51

[B25] Peterka R. J. (2002). Sensorimotor integration in human postural control. J. Neurophysiol. 88, 1097–1118. doi: 10.1152/jn.2002.88.3.1097 12205132

[B26] Raymakers J. A. Samson M. M. Verhaar H. J. (2005). The assessment of body sway and the choice of the stability parameter(s). Gait Posture 21, 48–58. doi: 10.1016/j.gaitpost.2003.11.006 15536033

[B27] Rose C. Edwards K. Siegler J. Graham K. Caillaud C. (2017). Whole-body cryotherapy as a recovery technique after exercise: A review of the literature. Int. J. Sports Med. 38, 1049–1060. doi: 10.1055/s-0043-114861 29161748

[B28] Ruhe A. Fejer R. Walker B. (2010). Center of pressure excursion as a measure of balance performance in patients with non-specific low back pain compared to healthy controls: A systematic review of the literature. Eur. Spine J. 20 (3), 358–368. doi: 10.1007/s00586-010-1543-2 20721676 PMC3048236

[B29] Santos M. J. Kanekar N. Aruin A. S. (2010). The role of anticipatory postural adjustments in compensatory control of posture: 2. Biomechanical analysis. J. Electromyogr Kinesiol 20, 398–405. doi: 10.1016/j.jelekin.2010.01.002 20156693 PMC2859839

[B30] Scoppa F. Capra R. Gallamini M. Shiffer R. (2013). Clinical stabilometry standardization: Basic definitions--acquisition interval--sampling frequency. Gait Posture 37, 290–292. doi: 10.1016/j.gaitpost.2012.07.009 22889928

[B31] Staszkiewicz R. Rusek S. Lubkowska A. Szyguła Z. (2025). Effect of whole-body cryotherapy on body balance in patients with multiple sclerosis. Acta Bioeng Biomech 27, 169–179. doi: 10.37190/abb-02584-2025-02 40544467

[B32] Surenkok O. Aytar A. Tüzün E. H. Akman M. N. (2008). Cryotherapy impairs knee joint position sense and balance. Isokinet Exercise Sci. 16, 69–73, 2-s2.0-41849129908. doi: 10.3233/ies-2008-0298

[B33] Taube W. Gruber M. Gollhofer A. (2008). Spinal and supraspinal adaptations associated with balance training and their functional relevance. Acta Physiol. 193, 101–116. doi: 10.1111/j.1748-1716.2008.01850.x 18346210

[B34] Williams E. E. Miller S. J. Sebastianelli W. J. Vairo G. L. (2013). Comparative immediate functional outcomes among cryotherapeutic interventions at the ankle. Int. J. Sports Phys. Ther. 8, 828–837. 24377069 PMC3867076

[B35] Winter D. (1995). Human balance and posture control during standing and walking. Gait Posture 3, 193–214. doi: 10.1016/0966-6362(96)82849-9

[B36] Winter D. A. Patla A. E. Prince F. Ishac M. Gielo-Perczak K. (1998). Stiffness control of balance in quiet standing. J. Neurophysiol. 80, 1211–1221. doi: 10.1152/jn.1998.80.3.1211 9744933

[B37] Zalewski P. Bitner A. Słomko J. Szrajda J. Klawe J. J. Tafil-Klawe M. . (2014). Whole-body cryostimulation increases parasympathetic outflow and decreases core body temperature. J. Therm Biol. 45, 75–80. doi: 10.1016/j.jtherbio.2014.08.001 25436954

[B38] Ziemann E. Olek R. A. Kujach S. Grzywacz T. Antosiewicz J. Garsztka T. . (2012). Five-day whole-body cryostimulation, blood inflammatory markers, and performance in high-ranking professional tennis players. J. Athletic Training 47, 664–672. doi: 10.4085/1062-6050-47.6.13 23182015 PMC3499891

